# Key Genes Involved in the Saline–Water Stress Tolerance of *Aloe vera*

**DOI:** 10.3390/cimb47121000

**Published:** 2025-11-28

**Authors:** María Mota-Ituarte, Jesús Josafath Quezada-Rivera, Aurelio Pedroza-Sandoval, Jorge Sáenz-Mata, Rafael Minjares-Fuentes

**Affiliations:** 1 Unidad Regional Universitaria de Zonas Áridas, Universidad Autónoma Chapingo, Bermejillo 35230, Mexico; mariamotaituarte@gmail.com (M.M.-I.); apedroza@chapingo.uruza.edu.mx (A.P.-S.); 2 Facultad de Ciencias Químicas, Universidad Juárez del Estado de Durango, Filadelfia, Gómez Palacio 35010, Mexico; 3 Facultad de Ciencias Biológicas, Universidad Juárez del Estado de Durango, Gómez Palacio 35010, Mexico; jsaenz_mata@ujed.mx

**Keywords:** *AOG* gene, *ABA2* gene, *GMMT* gene, *Aloe vera*, saline–water stress, mannose-rich polysaccharides

## Abstract

*Aloe vera* is well known for its high tolerance to adverse environmental conditions. However, the molecular pathways governing its adaptive response mechanisms to abiotic stress remain unclear. Thus, the expression of *AOG*, *ABA2*, and *GMMT* genes in *Aloe vera* plants subjected to saline–water stress was evaluated, with the expression of key genes significantly influenced by stress response. *AOG* and *GMMT* expression levels were higher under field capacity (FC) than under water deficit (PWP), with *AOG* reaching ~4.3% under 40 mM salinity at FC. In contrast, *ABA2* was strongly upregulated under PWP, particularly at 40 mM salinity, with expression increasing up to fivefold compared to the control. However, salinity above 40 mM led to reduced *ABA2* expression. *GMMT* was overexpressed (~6%) under severe stress, while mannose content increased significantly with salinity but remained unaffected by soil moisture. These findings highlight gene-specific responses to combined stress.

## 1. Introduction

Climate change is exerting unprecedented pressure on plant systems worldwide, challenging their survival through increased temperatures, altered precipitation patterns, and the intensification of abiotic stresses such as drought or salinity [1]. These environmental perturbations not only compromise plant productivity and biodiversity but also affect global food security and ecosystem resilience [2].

Among the species capable of withstanding such environmental adversity, *Aloe vera* (*A. vera*) has emerged as a model of physiological and biochemical resilience, displaying remarkable tolerance to drought and saline stress [2,3,4]. This succulent plant, native to arid and semi-arid regions, has demonstrated high adaptability to extreme conditions, which is likely to become increasingly prevalent under current climate scenarios [2,3,4,5,6,7,8,9,10,11]. Its survival strategy involves a suite of morphological and biochemical responses, including a reduction in the foliar area to limit evapotranspiration and enhanced synthesis of secondary metabolites, such as aloin, polysaccharides, and phenolic compounds, which collectively contribute to oxidative protection and osmotic balance [2,8].

A key component of *A. vera*’s stress tolerance is the biosynthesis of acemannan, a bioactive acetylated polysaccharide stored in the parenchymatous gel of the plant, which contributes to cellular water retention, membrane stabilization, and tissue elasticity [5,7,8,9,12]. This polysaccharide is synthesized by the enzyme glucomannan mannosyl-transferase (*GMMT*), encoded by the *CSLA9* gene, the expression of which is upregulated under abiotic stress [5,13]. Enhanced acemannan production under saline and drought conditions promotes cell-wall remodeling and pectin branching, improving mechanical stability and signal transduction during dehydration [8,9]. In parallel, *A. vera* activates a complex network of abscisic acid (ABA)-related genes, such as *ABA2* and *AOG*, which regulate ABA biosynthesis and conjugation, promoting stomatal closure and activating antioxidant defenses [1,5,14,15,16,17,18].

Despite these advances, the integrated relationship between water deficit, salinity, and the structural and functional behavior of acemannan remains insufficiently explored. Understanding these interactions is essential to elucidating the molecular basis of *A. vera*’s resilience and its potential application in developing climate-resilient crops. As global temperatures continue to rise, harnessing the intrinsic tolerance mechanisms of *A. vera*, either through metabolic engineering or adaptive breeding, may provide valuable strategies to sustain agricultural productivity in water-limited environments [19].

In this context, the present study aims to investigate the molecular and biochemical responses of *Aloe vera* under combined salinity and water-deficit stress, with a particular focus on the expression of key genes (*ABA2*, *AOG*, *GMMT*) involved in abscisic acid biosynthesis and acemannan polymerization. The findings of this study are expected to provide critical insights into the mechanisms underlying the plant’s resilience, contributing valuable knowledge for the development of abiotic stress-resistant crops adapted to arid and saline environments.

## 2. Materials and Methods

### 2.1. Plant Material

*Aloe vera* seedlings, aged 6 months and measuring between 25 and 30 cm, were used for the experiment. These seedlings were transplanted into 15 kg capacity pots filled with 10 kg of sandy loam soil collected from the study area. The experiment took place at the Regional University Unit for Arid Zones of the Autonomous University of Chapingo (Durango, Mexico) during the spring–summer of 2020. Prior to the application of stress conditions, all plants were subjected to standardized irrigation for four months as previously described by Mota-Ituarte et al. [2]. The irrigation treatments began when the Aloe plants reached ten months of age, corresponding to the juvenile or young plant stage. During this phase, and up to approximately one year of age, *Aloe vera* typically grows as a small, stemless rosette, concentrating its resources on root establishment and leaf expansion [20].

Five saline concentrations were tested at 0, 20, 40, 60, and 80 mM NaCl, along with two soil moisture levels: field capacity (FC) at 20.7% and the permanent wilting point (PWP) at 12.3% soil water content [2]. This setup resulted in ten treatments, including five salinity treatments irrigated at FC and five treatments under salinity and irrigated at PWP, with four repetitions of each treatment. Plants grown under 0 mM salinity and irrigated at FC were used as the control sample. All plants were irrigated weekly according to the experimental treatments for a period of 3 months. Soil moisture was monitored using an Extech Soil Moisture Meter MO750 (Nashua, NH, USA) [8]. It is important to note that *Aloe vera* has shown moderate salt tolerance, with most studies indicating that growth and gel yield are not affected at concentrations up to approximately 40 mM NaCl. However, negative effects on biomass, leaf development, and gel content become pronounced when salt concentrations exceed 60 mM NaCl [21,22,23,24].

After the stress treatments, the plants were carefully washed with distilled water using a spray bottle. For RNA extraction and subsequent RT-qPCR, the third leaf from the inside to the outside of the rosette of each plant was sectioned separately. The samples were immediately frozen in liquid nitrogen and stored at −70 °C until analysis.

### 2.2. RNA Extraction

RNA extraction was carried out using the exocarp or leaf cortex, separated from the parenchyma. This cortex was frozen with liquid nitrogen and ground with a mortar and pestle. This tissue was selected (1) to avoid contamination with plant mucilage, as polysaccharides can interfere with RNA extraction, and (2) because acemannan synthesis occurs in the parenchymal cells located in the middle region of the gel and cortex, from which it is subsequently transported to the inner part of the gel, where it functions as a storage polymer. This tissue was chosen to avoid contamination with plant mucilage, as polysaccharides interfere with RNA extraction. Thus, 700 µL of extraction buffer (2% *w*/*v* CTAB, 2% *w*/*v* PVP, 100 mM Tris-HCl, pH 8, 25 mM EDTA, 2 M NaCl, 0.05% spermidine) and 100 µL of β-mercaptoethanol were added to the sample. The mixture was vortexed at maximum speed for 30 s. Then, it was incubated for 10 min at 65 °C, inverting the tube four times. Subsequently, 500 µL of chloroform was added, and the mixture was vortexed again at maximum speed for 30 s, after which it was centrifuged at 10,000 rpm and 4 °C for 10 min. The supernatant was transferred to a new tube and 350 µL of phenol/chloroform/isoamyl alcohol (25:24:1) was added, following which the mixture was again vortexed at maximum speed for 30 s and centrifuged at 10,000 rpm and 4 °C for 10 min. The supernatant was transferred to a new tube, and an equal volume of chloroform/isoamyl alcohol (24:1) was added. The mixture was then vortexed for another 30 s and centrifuged at 10,000 rpm and 4 °C for 10 min. Again, the supernatant was transferred to a new tube, and 1/3 of the volume of 10 M LiCl was added. The sample was then left to precipitate overnight at 4 °C, after which it was centrifuged at 10,000 rpm and 4 °C for 20 min, decanted, and the sediment was washed with 800 µL of 96% ethanol. This mixture was then centrifuged at 10,000 rpm and 4 °C for 5 min. The sediment was then washed with 800 µL of 70% ethanol and centrifuged at 10,000 rpm and 4 °C for 5 min. Finally, the sediment was decanted, dried at room temperature, and resuspended in 20 µL of DEPC water.

Then, a DNase treatment was performed to degrade residual DNA using DNase I RNase-free (Ambion Life Technologies (Waltham, MA, USA) according to the provider’s instructions. Finally, the RNA extraction was evaluated for quantity, purity, and integrity using spectrophotometry (NanoDrop 2000, UV/Vis, Thermo Scientific, Waltham, MA, USA) and horizontal electrophoresis in 1% agarose gels.

### 2.3. Primers

A quantity of 40 ng of total RNA per reaction was used to estimate the expression of the *ABA 8H*, *ZEP*, *ABA2*, *AOG*, and *GMMT* genes, with *actin* as the reference gene, using specific primers (Table 1). All primers were synthesized by Sigma-Aldrich (Burlington, MA, USA).

### 2.4. RT-qPCR Quantitative Analysis

The RT-qPCR was conducted using the Power SYBR^®^ Green RNA-to-CT™ 1-Step Kit (ThermoFisher Scientific, Carlsbad, CA, USA) on a Step One™ Real-Time PCR System (Applied Biosystems, Carlsbad, CA, USA) according to the manufacturer’s protocol, with a reaction volume of 10 µL. A negative control, containing DEPC water, was included. The thermal cycling conditions were as follows: reverse transcription at 48 °C for 30 min, Taq polymerase activation at 95 °C for 10 min, initial denaturation at 95 °C for 15 s, and annealing at 60 °C for 1 min. This cycle was repeated 40 times, followed by a melting curve analysis: 95 °C for 15 s, 60 °C for 15 s, and 95 °C for 15 s. Relative expression levels were determined using the Quantitation–Comparative CT (ΔΔC_T_) method [26].

$$Fold\;change=2^{-∆∆C_{T}}$$

After RT-qPCR, melting curves were generated to verify the specificity of the amplification. Additionally, horizontal electrophoresis was performed on a 1.2% agarose gel as a complementary test. For this analysis, four biological replicates were used, and all assays were carried out in triplicate.

### 2.5. Biosynthesis of Mannose-Rich Polysaccharides

The biosynthesis of mannose-rich polysaccharides was associated with the mannose content. Thus, the extraction of mannose-rich polysaccharides, as well as the analysis of their carbohydrate composition, was conducted according to the methodology described by Comas-Serra et al. [9]. The insoluble alcohol residue was obtained from freeze-dried *A. vera* gel, which was then subjected to water extraction, while the water-soluble material was freeze-dried and subjected to hydrolysis. The released mannose was derivatized to its alditol acetate and quantified using a GC Hewlett-Packard 5890A (Waldbronn, BW, Germany) with an FID detector and equipped with a 30 m DB-225 column (J&W Scientific, Folsom, CA, USA) with an i.d. and a film thickness of 0.25 mm and 0.15 µm, respectively. The alditol acetates were isothermally separated at 220 °C. Mannose anhydride (99% purity, Sigma-Aldrich, Madrid, Spain) and 2-deoxy-glucose (99% purity, Sigma-Aldrich, Madrid, Spain) were used as the sugar and the internal standard, respectively [9].

### 2.6. Statistical Analysis

Prior to analysis, data normality was assessed using the Shapiro–Wilk test at a significance level of α > 0.05. Statistical analyses were conducted using ANOVA, considering soil moisture and NaCl concentration as factors. A General Linear Model (GLM) was applied to perform the ANOVA at a significance level of α < 0.05, and post hoc comparisons were carried out using the Least Significant Difference (LSD) test. All statistical analyses were performed in MINITAB^®^ software version 22.4.0 (State College, PA, USA).

## 3. Results

### 3.1. RNA Extraction and Quality

**Figure 1:**
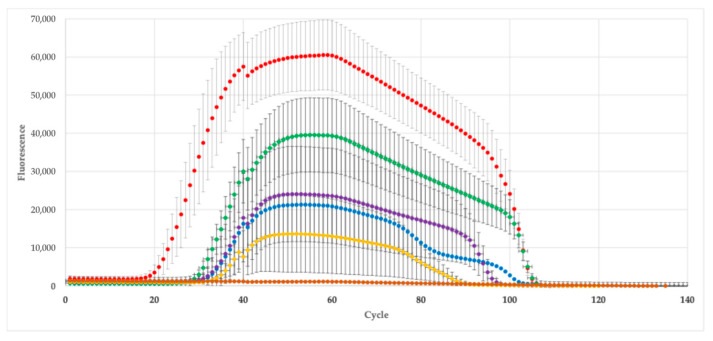
Amplification curves obtained from quantitative real-time PCR (qPCR) analysis showing fluorescence intensity as a function of cycle number. Each curve represents the amplification of the target genes (actin (•), *GMMT* (•), *AOG* (•), *ABA2* (•), *ABA8* (•), *ZEP* (•)), with error bars indicating the standard deviation of triplicate reactions.

The genes *ACT* (actin), *GMMT*, *AOG*, and *ABA2* showed well-defined amplification curves, with a clear exponential phase and high fluorescence levels, indicating good efficiency and specificity. Opposite to this, the *ABA8* and *ZEP* genes exhibited very low or flat signals, without reaching significant fluorescence levels, suggesting low expression (Figure 1). Thus, these last genes were excluded from further analysis.

### 3.2. Expression of Key Gens on Aloe vera Under Saline–Water Stress

#### 3.2.1. Expression of *AOG* in *Aloe vera* Under Saline–Water Stress

**Figure 2:**
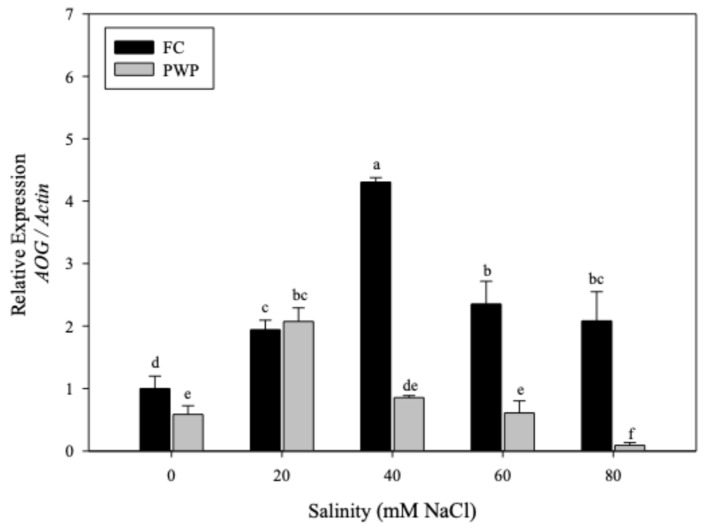
Relative expression of the *AOG* gene normalized to *Actin* in *Aloe vera* plants exposed to different NaCl concentrations (0–80 mM) under two soil moisture conditions: field capacity (FC, black bars) and permanent wilting point (PWP, gray bars). Data represent mean ± standard error (*n* = 4). Different letters indicate statistically significant differences among treatments according to the LSD test (*p* < 0.05).

The expression of the *AOG* gene in *A. vera* plants grown under saline–water stress is shown in Figure 2. As can be seen, the *AOG* expression was significantly affected by saline–water stress (*p* < 0.05). The *AOG* expression ranged from 0.1% to >4%, with the lowest expression being observed in those plants grown at higher stress conditions (PWP, −80 mM), whereas those plants grown under 40 mM salinity but irrigated at FC exhibited the highest expression of this gene. It is important to note that *AOG* expression was more significantly affected by water deficit than by saline stress, with most of the plants grown under water deficit exhibiting the lowest expression, at >1%. Interestingly, the plants in the control group exhibited a relative expression of the *AOG* gene of 1 ± 0.3%.

#### 3.2.2. Expression of *ABA2* in *Aloe vera* Under Saline–Water Stress

**Figure 3:**
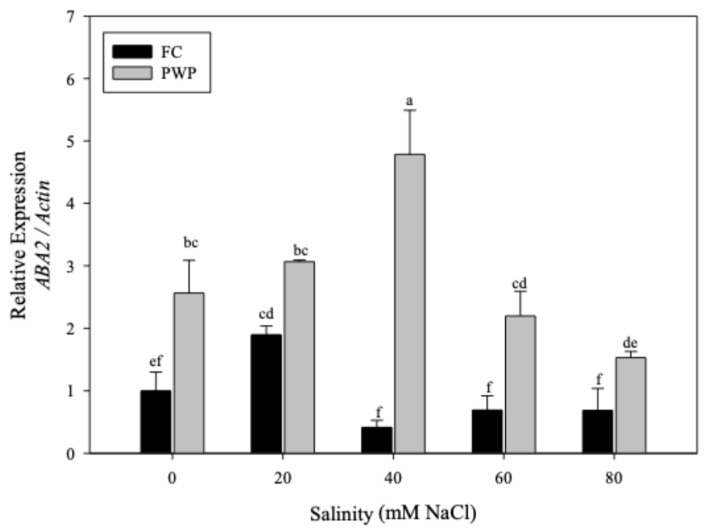
Relative expression of the *ABA2* gene normalized to *Actin* in *Aloe vera* plants exposed to different NaCl concentrations (0–80 mM) under two soil moisture conditions: field capacity (FC, black bars) and permanent wilting point (PWP, gray bars). Data represent mean ± standard error (*n* = 4). Different letters indicate statistically significant differences among treatments according to the LSD test (*p* < 0.05).

The relative *ABA2* expression showed significant differences between treatments (*p* < 0.05) (Figure 3). Interestingly, this gene exhibited higher expression than *AOG* when *A. vera* plants were subjected to salinity–water stress, reaching expression levels around 5.5%. Furthermore, the plants grown at field capacity (FC) conditions exhibited lower *ABA2* expression than the plants under permanent wilting point (PWP) conditions. Notably, the plants grown under PWP conditions and 40 mM salinity showed positive regulation, denoted by expression levels increasing approximately fourfold compared to the control samples. Curiously, the lowest levels of *ABA2* were observed when *Aloe vera* was grown under higher-salinity conditions. Specifically, *ABA2* levels were reduced to less than 2% when salinity exceeded 40 mM at PWP and dropped to below 1% when salinity was greater than 20 mM at FC (*p* < 0.05).

#### 3.2.3. Expression of *GMMT* in *Aloe vera* Under Saline–Water Stress

**Figure 4:**
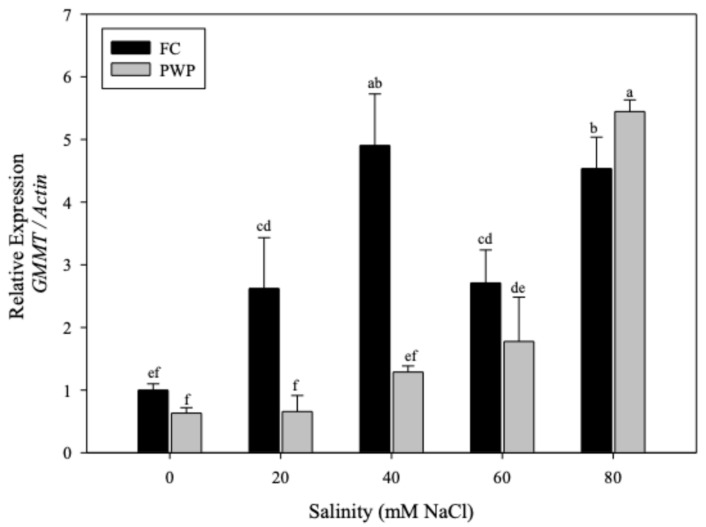
Relative expression of the *GMMT* gene normalized to *Actin* in *Aloe vera* plants exposed to different NaCl concentrations (0–80 mM) under two soil moisture conditions: field capacity (FC, black bars) and permanent wilting point (PWP, gray bars). Data represent mean ± standard error (*n* = 4). Different letters indicate statistically significant differences among treatments according to the LSD test (*p* < 0.05).

Saline–water stress promoted the overexpression of glucomannan mannosyl transferase (*GMMT*) in *A. vera* (see Figure 4). The expression of *GMMT* in *Aloe* plants grown under FC was higher than that of plants grown under PWP, except for the plants exposed to the most extreme conditions (PWP with 80 mM salinity) (*p* < 0.05). Interestingly, a significant increase in the *GMMT* levels was observed in the plants grown under 40mM salinity and FC. However, further increases in salinity led to a significant reduction in *GMMT* expression. Meanwhile, *Aloe vera* grown under PWP conditions reached the highest *GMMT* levels at 80 mM, the most severe condition.

### 3.3. Effect of Salinity and Water Stress on Gene Expression

Table 2 presents the results of the analysis of variance (ANOVA) regarding the effects of soil moisture level, salinity, and their interaction on the expression of the *GMMT*, *ABA2*, and *AOG* genes. All factors showed statistically significant effects (*p* < 0.05) on gene expression. Notably, the interaction between soil moisture and salinity exhibited a significant influence on the expression of all three genes (*GMMT*: F = 14.82, *ABA2*: F = 17.07, *AOG*: F = 96.28), indicating that the combined stress conditions modulate their transcriptional response more strongly than individual factors alone.

### 3.4. Biosynthesis of Mannose-Rich Polysaccharides in Aloe vera

**Figure 5:**
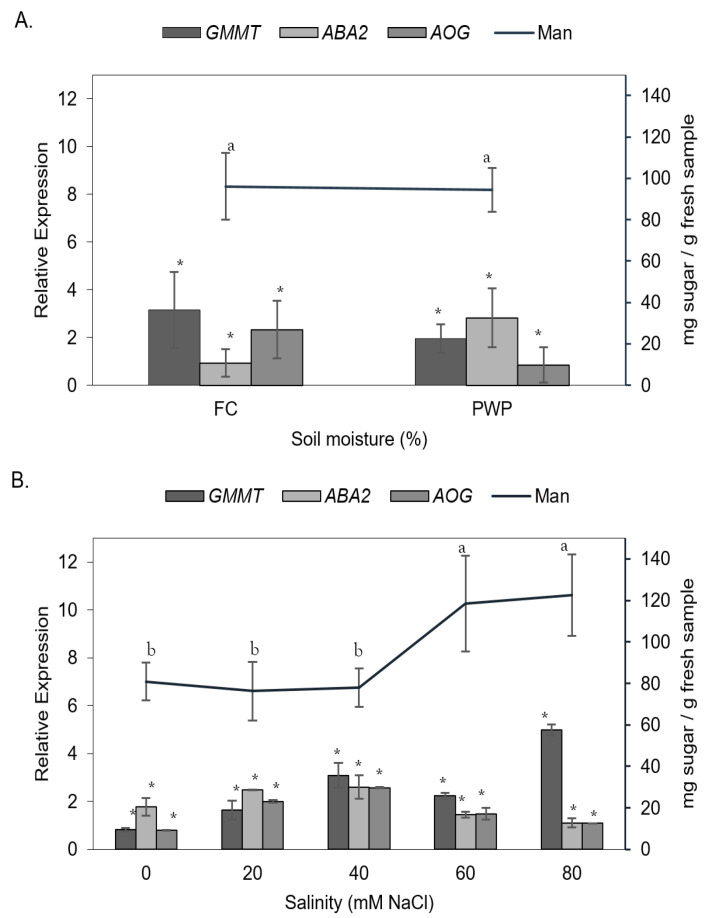
Relative expression of *GMMT*, *ABA2*, and *AOG* genes and mannose content (Man) in *Aloe vera* leaves under different abiotic stress conditions. (**A**) Effect of soil moisture: field capacity (FC) and permanent wilting point (PWP). (**B**) Effect of salinity at different NaCl concentrations (0–80 mM). Bars represent the relative expression levels (mean ± SD, *n* = 4) of the analyzed genes, while the line indicates mannose content (mg sugar /g fresh sample). Different lowercase letters above the line indicate significant differences (*p* < 0.05) in mannose content among treatments, while asterisks (*) denote significant differences (*p* < 0.05) in gene expression compared with the control.

Mannose-rich polysaccharides play a key role in the stress tolerance of *A. vera* plants. Thus, the content of mannose as a function of individual stressors, namely water deficit and saline concentration, was evaluated (Figure 5). As can be seen, the mannose content was not significantly affected by soil moisture levels (FC or PWP, *p* > 0.05). However, the salinity led to a significant increase in mannose level, from ~60 up to ~140 mg/g sample, in the parenchymatous tissue (*p* < 0.05). On the other hand, the effect of saline–water stress on gene expression in *A. vera* was also compared based on individual stressors.

The expression of *AOG* and *GMMT* genes was higher in plants grown under FC conditions than in those grown under PWP conditions. Notably, the *ABA2* gene was overexpressed when *A. vera* was subjected to water restriction (PWP condition). Interestingly, the expression of these genes increased as salinity increased from 0 to 40 mM NaCl but decreased when salinity exceeded 40 mM, except for the *GMMT* gene, which was overexpressed by nearly 6%. Mannose has been recognized as a characteristic fingerprint of the acemannan polymer in *Aloe vera* [3,4]. Therefore, high mannose levels indicate a greater abundance of acemannan. Within this context, the elevated mannose content observed under the most severe stress conditions suggests an increased accumulation of acemannan. This finding suggests that the *GMMT* gene may have undergone overexpression, leading to enhanced production of the enzyme responsible for linking mannose units to the main backbone of the acemannan polymer.

## 4. Discussion

*Aloe vera* (*Aloe barbadensis* Miller) is, without doubt, one of the most popular plants used in traditional medicine and, recently, in the food industry. This popularity has largely been attributed to its high adaptability to adverse environments, including drought and saline conditions. However, despite its high tolerance to adverse conditions, *A. vera* can still experience abiotic stress when a water deficit and salinity occur simultaneously [6,8,9]. *Aloe vera*’s tolerance to water and salt stress appears to be closely linked to the regulation of specific biosynthetic pathways mediated by abscisic acid (ABA), particularly those involved in the synthesis of acemannan, a functionally important acetylated glucomannan [5]. Several studies have shown that ABA not only acts as a physiological regulator in response to water deficit but also directly modulates gene expression, promoting the synthesis of hydrophilic polysaccharides capable of retaining water in parenchymal tissues [14,27,28]. In this context, the *CSLA9* gene, which encodes the enzyme glucomannan mannosyltransferase (*GMMT*), has been identified as a central element in the synthesis of the acemannan skeleton [5]. Previously, Salinas et al. [5] conducted a study on the effect of water deficit and exogenous abscisic acid (ABA) application on the regulation of the *GMMT* gene in *Aloe vera*. They observed that water deficit played a significant role in regulating this gene, particularly when plants were irrigated at 50% of field capacity, while its expression was suppressed under severe stress conditions. Additionally, they applied a single dose of exogenous abscisic acid (10 mM) to plants not subjected to water deficit and found that the *GMMT* gene exhibited two peaks of overexpression, with the first being within the initial 48 h after application and the second being between the first and second week, confirming that ABA plays an important role in the regulation of *GMMT*. They speculated that the residence time of ABA within the plant might be sufficiently long to induce the activation of transcription factors at different time points, thereby positively regulating *GMMT* expression. At the metabolic level, a significant accumulation of acemannan has been observed under combined stress conditions (water deficit and salinity), along with an increase in its acetylation level, suggesting a structural adaptation to enhance water retention [8,9]. In parallel, endogenous ABA levels progressively increase with stress severity, reaching increases of up to 15.5-fold compared to the control, reinforcing its role as a molecular signal in this response [5]. At this point, it is important to note that *GMMT* activation does not occur in isolation, as the genes *AOG* and *ABA2*, involved in the transport of conjugated ABA with glucose ester (ABA-GE) and its conversion into active ABA, respectively, show expression patterns consistent with stress levels. Interestingly, Salinas et al. [5] reported a pronounced increase in ABA-derived metabolites under water-deficit conditions, with ABA-GE exhibiting the most substantial accumulation. Even at the highest stress intensity, ABA-GE concentrations increased from approximately 100 to 800 pmol/g DW. This marked elevation suggests that the conjugated, inactive form of ABA may serve as a reliable metabolic marker of water stress in *Aloe vera*. Consistent with these observations, abiotic stress is known to activate ABA biosynthesis, catabolism, and transport pathways, collectively inducing the transcription of genes associated with ABA metabolism and signaling. Notably, ABA biosynthesis involves the coordinated expression of several key genes, including *ZEP* and *ABA8*, which play essential roles in the regulation of ABA homeostasis under stress conditions [29]. Interestingly, it has been well established that the zeaxanthin epoxide (*ZEP*) gene exerts a regulatory function in the biosynthesis of ABA. Several studies have demonstrated that species such as *Nicotiana plumbaginifolia*, *Arabidopsis thaliana*, and *Lycopersicon esculentum* exhibit an upregulation of *ZEP* expression at the onset of drought stress, supporting its central role in the modulation of ABA synthesis [30,31]. Nevertheless, Barrero et al. [32] proposed that ABA biosynthesis may also be governed by alternative regulatory pathways independent of *ZEP* activity. In the present study, the combined effects of salinity and water deficit may have contributed to the low amplification of the *ZEP* gene, whereas the more severe abiotic stress conditions likely resulted in its transcriptional suppression. Based on these observations, it can be hypothesized that the overexpression of the *ZEP* gene occurs primarily during the initial phases of stress perception and that prolonged or intensified stress may downregulate its expression as part of a broader adaptive response. On the other hand, the *ABA8* gene belongs to a family of genes involved in the catabolic process of ABA, with this gene being the predominant member of the family. The main function of such genes is to catabolize excess ABA produced by the plant grown under normal, non-stressed conditions. In this context, the low fluorescence signal of the *ABA8* gene may indicate, on one hand, a low ABA content associated with the reduced expression of the *ABA2* gene, and, on the other hand, may suggest the activation of an alternative survival mechanism, whereby the plant prioritizes stress endurance over productivity under extreme abiotic stress conditions. Meanwhile, *ABA2* was especially sensitive to water deficits, exhibiting strong overexpression under PWP conditions and 40 mM salinity, followed by a marked decline. The highest expression could be attributed to the ability of *Aloe vera* to metabolize the ABA-GE into ABA in order to sustain gel production. However, under the most severe stress conditions, the reduced expression of *ABA2* suggests a possible shift in the metabolic pathway, enabling the plant to maintain a latent physiological state. This could be associated with the findings reported by Mota-Ituarte et al. [2] and González-Delgado et al. [8], who assessed the morphology and appearance of *A. vera* plants under salinity and water stress. They noted that those plants exposed to the most severe stress conditions exhibited a closed rosette structure, along with leaf wedging, grooving, and apex necrosis, accompanied by a marked reduction in fresh biomass, which remained nearly constant (~1.2 kg per plant) at salinity levels above 40 mM under PWP conditions. For its part, *AOG* showed differential regulation, decreasing drastically under high combined stress conditions, suggesting a limitation in the intracellular availability of active ABA under extreme conditions. This gene catalyzes the conversion of ABA into its conjugated form, ABA-GE, thereby regulating the levels of ABA and maintaining hormonal homeostasis [14]. Within this context, the suppression of the *AOG* gene under severe stress conditions may indicate a shift in the ABA metabolic balance, leading to the conversion of ABA into its inactive conjugated form, ABA-GE. This process could represent a protective mechanism to prevent the excessive accumulation of active ABA, thereby avoiding prolonged stress signaling. Alternatively, ABA-GE may be stored only transiently, as its synthesis and conjugation require substantial energy investment, which is often limited under extreme stress. This behavior suggests that *A. vera* modulates its ABA homeostasis to optimize energy use and maintain metabolic stability, contributing to its adaptive response and survival under harsh environmental conditions. Together, these findings highlight the ABA-GE → ABA activation pathway mediated by *AOG* and *ABA2* as a key regulatory mechanism of *GMMT* expression. The overexpression of the latter under stress suggests an adaptive mechanism through which *A. vera* enhances acemannan biosynthesis as part of its osmotic adjustment strategy. This response likely contributes to counteracting water loss and preserving the functional integrity of the gel, which acts as an intrinsic water reservoir and plays a crucial role in maintaining cellular hydration and tissue stability under adverse environmental conditions. From a biochemical perspective, stress conditions also alter cell wall polysaccharides. Mannose content increased significantly under salinity, reinforcing the connection between *GMMT* regulation and mannose-rich polysaccharide accumulation. It is important to highlight that water deficit alone induces both quantitative and qualitative modifications in acemannan, revealing an adaptive metabolic response to drought [7]. In fact, it has been reported that reducing irrigation from 100% (D0) to 60% (D60) of field capacity caused the mannose content to decline from 175.1 to 102.9 mg/g [7]. Despite the reduction in total polysaccharide yield, acemannan preserved its acetylation degree (~1.0 acetyl group per mannose), whereas its molecular weight nearly doubled (~54 → 98 kDa), suggesting selective degradation of smaller chains and the retention of larger, highly acetylated fractions with stronger water-binding capacity [7]. From a rheological point of view, these structural modifications resulted in increased viscosity (0.12 → 0.28 Pa·s) and greater pseudoplasticity (*n* = 0.57 → 0.47), along with weak-gel behavior evidenced by a G′/G″ crossover. These findings indicate that a water deficit triggers a molecular reorganization of acemannan, enhancing its viscoelastic and hydration-retaining properties to preserve gel integrity and sustain water balance within the parenchymal tissues of *Aloe vera* under drought conditions [7]. Interestingly, the combination of salinity and water deficit induces notable compositional and structural modifications in acemannan, closely linked to morphological and functional responses [2,8,9]. Under conditions with low soil moisture levels and increasing salinity (up to 80 mM NaCl), plants exhibited leaf folding, wedging, and apex necrosis, with reduced leaf width and biomass, reflecting morphological adaptations to minimize water loss [2,8]. At the molecular level, the mannose content increased from ~194 to 345 mg/g WSP, and the acetylation degree rose by 90–150%, indicating the synthesis of highly acetylated, high-molecular-weight acemannan fractions with enhanced water-binding capacity [9]. These features, together with the high degree of pectin methyl-esterification (~60%), contribute to stabilizing the cell wall and preventing structural collapse [8,9], supporting the hypothesis that acemannan and pectins play a central role in the resilience strategy of *A. vera* under stress conditions [9].

## 5. Conclusions

The present study aimed to explore the expression of *GMMT* and other genes involved in the biosynthesis of abscisic acid, such as *ABA2*, *AOG*, *ABA8*, and *ZEP*, under combined salinity and water deficit conditions, as well as their relationship with the synthesis of the acemannan polymer. This research holds relevance because (1) soil conditions in arid and semi-arid regions are becoming increasingly unfavorable due to the combined effects of water scarcity and elevated salinity, and (2) many of the functional attributes traditionally ascribed to *A. vera* are directly linked to the structural and physicochemical properties of the acemannan polymer. Overall, these findings suggest that *A. vera* regulates its ABA metabolic network through a dynamic, stress-dependent modulation of key biosynthetic and catabolic genes. The overexpression of *ZEP* appears to occur primarily during the early stages of stress perception, whereas its suppression under prolonged or intensified stress likely reflects a broader adaptive strategy to modulate ABA synthesis. Likewise, the weak fluorescence signal of *ABA8*, combined with the reduced expression of *ABA2* under severe stress, indicates both a diminished ABA pool and the possible activation of alternative survival pathways that prioritize stress tolerance compared to growth. Similarly, the downregulation of *AOG* under extreme stress conditions suggests a shift toward ABA conjugation as a protective mechanism to prevent the excessive accumulation of active ABA, thereby avoiding prolonged stress signaling. In contrast, the strong induction of *GMMT* underscores its central role in enhancing acemannan biosynthesis, contributing to osmotic adjustment and maintaining gel integrity as a water-retaining matrix. Collectively, these coordinated transcriptional and biochemical adjustments highlight the capacity of *A. vera* to finely regulate its hormonal homeostasis and polysaccharide metabolism, ensuring metabolic stability and cellular hydration essential for survival under severe abiotic stress conditions. Further studies are necessary to expand the analysis of the genes that showed low or minimal expression in the present work, allowing for a more comprehensive characterization of their functions within the stress-response network. Likewise, these studies will enable improved traceability of the transcription factors regulating these genes through the implementation of continuous, time-resolved monitoring throughout the progression of stress, rather than relying solely on end-point measurements. This approach might provide a more detailed understanding of the temporal dynamics governing the molecular response of *A. vera* to abiotic stress.

## Figures and Tables

**Table 1:** Primers sequences, melting temperatures, and amplicon sizes for genes involved in ABA biosynthesis and cell-wall polysaccharide metabolism in *Aloe vera*.

Gene	Encoded Enzyme	Primer Sequence	Melting Temperature (°C)	Amplicon Size (bp)	Reference
*ABA 8H-F*	Abscisic acid 8′-hydroxylase	5′-GAGAGAGAGAGGTGCTACATTTG-3′	54.0	206.0	[25]
*ABA 8H-R*		5′-ATGTTTGGGTCTTGAGAGTAGAG-3′			
*ZEP-F*	Zeaxanthin epoxidase	5′-GAAACTTGGGCAAAGGGAATG-3′	55.0	258.0	[25]
*ZEP-R*		5′-CTTGTTGTACCCACCCTGATAG-3′			
*ABA2-F*	Zeaxanthin epoxidase chloroplastic	5′-GGACAGTACAGAGGTCCAATTC-3′	55.0	333.0	[25]
*ABA2-R*		5′-TCCTCAGCAACCTCCAAATC-3′			
*AOG-F*	Abscisate beta- glucosyltransferase	5′-GGTGCCCACCCTCTTATTATC-3′	54.0	277.0	[25]
*AOG-R*		5′-AAGGTGAAGGAGGAGGAGAA-3′			
*GMMT-F*	Glucomannan mannosyltransferase	5′-GTCCAGATCCCCATGTTCAACGAG-3′	60.0	107.0	[5]
*GMMT-R*		5′-CCAACAGAATTGAGAAGGGTGAT-3′			
*Act-F*	Actin	5′-AGCCGTCGATGATTGGGATG-3′	60.0	116.0	[5]
*Act-R*		5′-CCACTGAGCACAATGTTGCC-3′			

**Table 2:** Analysis of variance (ANOVA) results regarding the effects of soil moisture level, salinity, and their interaction on *GMMT*, *ABA2*, and *AOG* expression.

	*GMMT*		*ABA2*		*AOG*	
	*F*	*p*	*F*	*p*	*F*	*p*
Soil moisture level	36.33	<0.0001	152.14	<0.0001	539.93	<0.0001
Salinity	51.83	<0.0001	14.27	0.001	99.48	<0.0001
Interaction	14.82	<0.0001	17.07	0.0006	96.28	<0.0001

## Data Availability

The original contributions presented in this study are included in the article. Further inquiries can be directed to the corresponding author.
